# Complete Genome Sequence of *Lactococcus lactis* subsp. *lactis* KLDS4.0325

**DOI:** 10.1128/genomeA.00962-13

**Published:** 2013-11-27

**Authors:** Xiaochun Yang, Yutang Wang, Guicheng Huo

**Affiliations:** Key Laboratory of Dairy Science (Ministry of Education), Northeast Agricultural University, Harbin, China

## Abstract

We report the complete genome sequence of *Lactococcus lactis* subsp. *lactis* KLDS4.0325, a probiotic bacterium isolated from homemade koumiss in Xinjiang, China. We have determined the complete genome sequence of strain KLDS4.0325, which consists of a chromosome and three plasmids and reveals genes that are likely to be involved in dairy fermentation and that have probiotic qualities.

## GENOME ANNOUNCEMENT

*Lactococcus lactis* is widely used in the production of fermented food products, such as yogurt and cheese. Some *L. lactis* strains have been extensively characterized functionally to document their probiotic attributes (1–8). The *L. lactis* strains are subdivided into two lineages, *L. lactis* subsp. *cremoris* and *L. lactis* subsp. *lactis*, based on their genotypes and phenotypes. The strain *L. lactis* subsp. *lactis* KLDS4.0325 was isolated in 2007. It was shown to have a characteristic pattern of high-yield l-lactic acid, to produce folate and riboflavin, and to possess antibacterial and antifreezing properties. The complete genome sequence of strain KLDS4.0325 was determined by whole-genome shotgun sequencing, using Sanger technology, of a clone library with an insert size of 500 bp. The genome was assembled using the SOAP*denovo* software (9), and multiplex PCR was used to close the gaps and remove regions of low coverage (10). The software program Glimmer (11) and the RAST suite (12) were used to identify protein-coding genes and for gene annotation, respectively.

The complete genome of strain KLDS4.0325 contains a single circular chromosome of 2,589,261 bp and three plasmids (plasmid 1 [5.7 kb], plasmid 2 [2.1 kb], and plasmid 3 [2.7 kb]). The overall G+C content of the chromosome is 35.4%, with 2,662 predicted open reading frames (ORFs), of which 1,310 were functionally classified. Sixty-two tRNA genes and 6 rRNA genes were also found. The G+C content of plasmids is 34.7%, with 212 predicted ORFs (13).

A comparative analysis of strain KLDS4.0325 and four other *L. lactis* genomes was performed using MUMmer (14), SplitTree4 (15), and Mauve (16). Strain KLDS4.0325 shares 2,438 ORFs with *L. lactis* subsp. *lactis* IL1403*, L. lactis* subsp. *lactis* CV56, *L. lactis* subsp. *lactis* KF147, and *L. lactis* subsp. *cremoris* NZ9000, and 2,215 ORFs have 80% sequence identity. A comparative genomics approach was performed to analyze proteolytic systems, the metabolic pathways of amino acids, and the genes involved in the production of lactic acid, vitamin B complex, bacteriocin, and cold stress proteins. The results show that not only can this strain hydrolyze extracellular proteins, transport, and perform enzymolysis efficiently, but the strain has the more complete enzyme systems of transamination and the deamination pathway. Therefore, the strain can metabolize related proteins and produce a series of flavor compounds. Otherwise, the strain possesses more key enzyme-coding genes involved in transport, sugar metabolism, and synthesis for l-lactic acid, folate, and riboflavin, and it has a gene cluster for wool sulfur antibiotic and two genes of cold stress proteins CspD and CspE. In addition, in a plasmid of strain KLDS4.0325, We found the bacteriocin-synthesis genes *lsbA* and *lsbB* and one hyaluronan synthase gene that has never been reported in other *Lactococcus* species; its homology with *Enterococcus faecalis* ATCC 29200 is up to 95%. The presence of these genes encoding desirable traits that provide the theoretical basis for the strain can help in industrial fermentation, which has good commercial value.

### Nucleotide sequence accession numbers.

The complete genome of *L. lactis* subsp. *lactis* KLDS4.0325 has been deposited in GenBank under accession no. CP006766 (chromosome) and CP006767 (plasmid 1).
